# ME1 promotes basal-like breast cancer progression and associates with poor prognosis

**DOI:** 10.1038/s41598-018-35106-y

**Published:** 2018-11-13

**Authors:** Ruocen Liao, Guoping Ren, Huixin Liu, Xingyu Chen, Qianhua Cao, Xuebiao Wu, Jun Li, Chenfang Dong

**Affiliations:** 1 Department of Pathology and Pathophysiology, and Department of Surgical Oncology (breast center) of The Second Affiliated Hospital, Zhejiang University School of Medicine, Hangzhou, 310058 China; 2 Zhejiang Key Laboratory for Disease Proteomics, Zhejiang University School of Medicine, Hangzhou, 310058 China; 3 Department of Pathology, The First Affiliated Hospital, Zhejiang University School of Medicine, Hangzhou, 310058 China

## Abstract

Basal-like breast cancer (BLBC) is associated with a poor clinical outcome due to the few treatment options and absence of effective targeted agents. Here, we show that malic enzyme 1 (ME1) is dramatically upregulated in BLBC due to ME1 copy number amplification. ME1 expression increases glucose uptake and lactate production, and reduces oxygen consumption, leading to aerobic glycolysis. ME1 expression promotes, whereas knockdown of ME1 expression suppresses tumorigenicity. In breast cancer patients, ME1 expression is positively correlated with large tumor size, high grade, poor survival, and chemotherapy resistance. Our study not only contributes to a new understanding of how metabolic reprogramming contributes to BLBC progression, but also provides a potential prognostic marker and therapeutic target for this challenging disease.

## Introduction

Breast cancer is a heterogeneous disease. According to gene expression profiling, breast cancer is divided into four biologically different subtypes: luminal A, luminal B, Her2-overexpressing, and basal-like subtypes. This classification also is reproduced to a certain extent in histologic subtypes^1^. Compared to the other subtypes, patients with basal-like breast cancer (BLBC) show an aggressive clinical history, resistance to chemotherapy, early recurrence, distant metastasis and poor prognosis^2–5^. BLBC expresses molecular markers that are characteristic of myoepithelial/basal cells, which is preferentially negative for estrogen receptor (ER), progesterone receptor (PR) and Her2 receptor (i.e. triple-negative), and positive for basal markers (EGFR, CK17, CK5/6 and CK14)^3,6^. BLBC has poor response to chemotherapy and lacks the targeted therapy, such as anti-ER and anti-Her2^7,8^. Therefore, a better understanding of BLBC and identifying the relevant targets are urgently needed.

Tumor cells preferentially metabolized glucose to lactic acid even in the presence of oxygen, which is known as aerobic glycolysis or the Warburg effect^9^. Aerobic glycolysis contributes to enhanced glucose consumption, lactate production and biosynthesis of macromolecules as well as reduced OXPHOS, and has been shown to be driven by hypoxia, oncogenic stimuli, mitochondrial defects, and aberrantly elevated glycolytic gene expression^9–13^. Particularly, elevated expression of glycolytic enzymes is widely reported in a variety of cancers, including breast, prostate, lung and colon. Some oncogenes such as c-MYC, mTOR and Snail activate glycolytic flux by upregulating expression of glycolysis enzymes including hexokinases and phosphofructokinase 1 (PFK1), and/or downregulating expression of gluconeogenesis enzymes such as fructose-1,6-biphosphatase (FBP1)^14–16^. Conversely, the tumor suppressor p53 can suppress tumor progression by inhibiting glycolytic flux NADPH production^17^. Thus, the Warburg effect is a vital component of the metabolic reprogramming and provides potential metabolic and survival advantages in cancer progression.

Malic enzyme 1 (ME1) is a cytosolic, NADP-dependent enzyme that catalyzes the oxidative decarboxylation of malate to pyruvate and reduces NADP^+^ to NADPH. ME1 expression is upregulated in human cancers, such as nasopharyngeal carcinoma and neuroblastoma. ME1 promotes tumor progression by interacting with p53^18^. Downregulation of ME1 inhibits migratory and invasive abilities of nasopharyngeal cancer cells^19^. These results clearly suggest that ME1 plays an important role in modulating tumor progression. Although there is evidence to support an oncogenic role for ME1, its contribution is poorly studied in breast cancer and its distinct subtypes. In this study, we investigated the function and underlying mechanism of ME1 in BLBC.

## Results

### ME1 is upregulated in BLBC subtype

**Figure 1:**
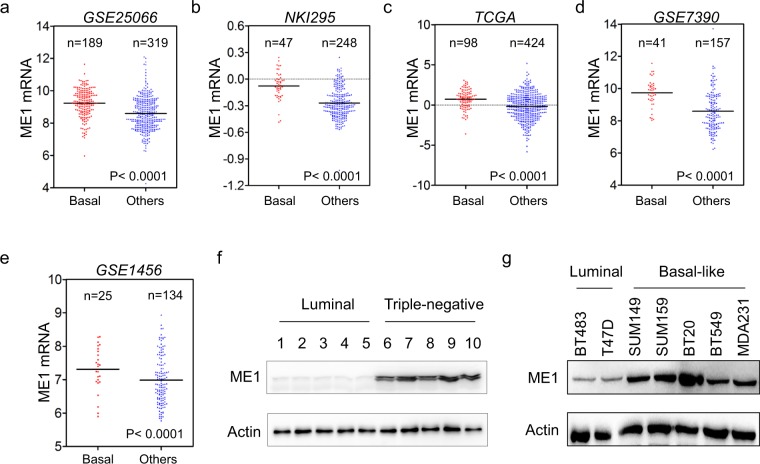
ME1 expression is upregulated in BLBC. (**a**–**e**) ME1 expression was obtained from five publicly available gene expression datasets, including GSE25066 (**a**), NKI295 (**b**), TCGA (**c**), GSE7390 (**d**) and GSE1456 (**e**). Scatter dot-plots indicated ME1 expression in basal-like and other subtype of breast cancer from these datasets. (**f**) Expression of ME1 was examined by western blotting in tumor samples from five cases of luminal and five cases of triple-negative breast cancer. (**g**) Expression of ME1 in two luminal and five BLBC cell lines was analyzed by western blotting.

We recently reported several metabolic genes aldo-keto reductase 1 member B1 (AKR1B1), fructose-1,6-biphosphatase (FBP1) and urine diphosphate–galactose ceramide galactosyltransferase (UGT8) that contributed to BLBC progression^14,20,21^. To identify other metabolic genes associated with BLBC, we analyzed gene expression profiles of breast cancer in five publicly available gene expression datasets (GSE25066, NKI295, TCGA, GSE7390 and GSE1456), which contain: 508, 295, 522, 198 and 159 breast cancer patients, respectively^22–25^. We noticed that ME1 expression was significantly higher in BLBC than in other subtypes. (Fig. 1a–e). To confirm this observation, we collected fresh frozen breast tumor tissues from 5 cases of luminal subtype and 5 cases of triple-negative subtype that is mostly BLBC. Indeed, ME1 expression was elevated in triple-negative breast cancer, and significantly downregulated in luminal subtype of breast cancers (Fig. 1f and Supplementary Fig. 1a). To further extend this finding, we analyzed ME1 expression in two luminal and five BLBC cell lines. We observed that ME1 expression also was high in BLBC cell lines, whereas it was low in luminal cell lines (Fig. 1g and Supplementary Fig. 1b). These findings indicate that ME1 expression is primarily enriched in BLBC.

### ME1 copy number amplification positively associates with ME1 expression and BLBC subtype

**Figure 2:**
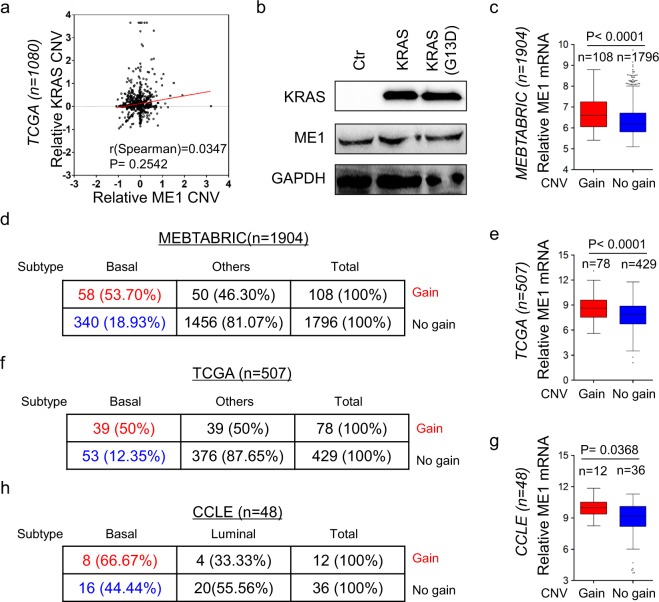
ME1 copy number amplification associates with ME1 expression and BLBC subtype. (**a**) Analysis of TCGA dataset for the CNVs of ME1 and TCGA. The relative CNV of ME1 was plotted against that of KRAS. Correlations were analyzed using Spearman’s rank correlation test. (**b**) Expression of ME1 and KRAS in T47D cells was analyzed by western blotting. (**c**) Box-plots indicated the association of ME1 expression with its copy number status (gain or no gain) in breast cancer from MEBTABRIC dataset. (**d**) Analysis of MEBTABRIC dataset for the association of copy number status of ME1 and tumor subtype. (**e**) Box-plots indicated the association of ME1 expression with its copy number status (gain or no gain) in breast cancer from TCGA dataset. (**f**) Analysis of TCGA dataset for the association of copy number status of ME1 and tumor subtype. (**g**) Box-plots indicated the association of ME1 expression with its copy number status (gain or no gain) in breast cancer from CCLE dataset. (**h**) Analysis of CCLE dataset for the association of copy number status of ME1 and tumor subtype.

KRAS regulates the expression of GOT1 and GLUD that acts upstream of the same pathway as ME1^26^. Moreover, KRAS expression has been shown to be important in maintaining the mesenchymal features of BLBC^27^. Thus it is possible that KRAS might associate with ME1 expression in BLBC. We first analyzed the CNVs’ correlation between KRAS and ME1 in the TCGA dataset, showing that no significant correlation was observed between both genes (Fig. 2a).We also examined the expression of ME1 by western blotting in T47D cells transfected with empty vector, KRAS-expressing vector or oncogenic KRAS (G13D)-expressing vector. Unexpectedly, KRAS or oncogenic KRAS (G13D) expression didn’t significantly cause a change in ME1 expression (Fig. 2b). These data indicate the involvement of other biological events.

Copy Number Variants (CNVs) of protein coding genes is associates with gene expression changes. To investigate the effect of CNVs on ME1 expression, we analyzed copy number alterations of breast cancer in two publicly available datasets (MEBTABRIC and TCGA), which contain: 1904 and 507 breast cancer patients, respectively. We observed that ME1 was amplified in breast cancer, with 5.7% and 15.4% of primary breast tumors having ME1 amplification in MEBTABRIC dataset and TCGA dataset, respectively (Fig. 2c–f). Notably, 53.7% and 50% of ME1 copy number amplification fell into BLBC subtype (Fig. 2d,f). Consistently, analysis of patient-derived breast cancer cell lines (CCLE dataset) showed that 25% (12/48) of cell lines have ME1 copy number amplification (Fig. 2g), 66.67% (8/12) of which belonged to BLBC subtype (Fig. 2h). In these datasets examined, ME1 expression was positively correlated with copy number amplification of ME1 (Fig. 2c–h). These data strongly associate ME1 copy number amplification with ME1 overexpression and BLBC subtype.

### ME1 contributes to aerobic glycolysis of breast cancer cells

**Figure 3:**
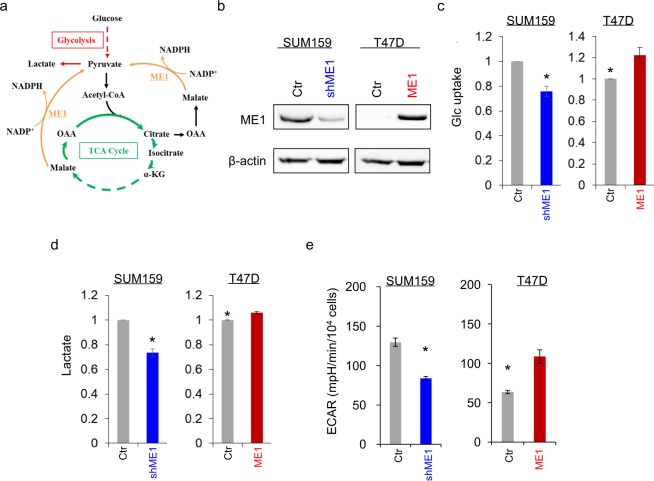
ME1 induces cell glucose uptake and lactate production. (**a**) Schematic diagram showing ME1-mediated metabolic pathways. (**b**) Stable transfectants with empty vector or knockdown of ME1 expression were established in SUM159 cells, and stable clones with empty vector or ME1 expression were generated in T47D cells. ME1 expression in these cells was examined by western blotting. Actin was used as a loading control. (**c**) The level of glucose uptake was measured in SUM159 cells with stable empty vector or knockdown of ME1 expression (left panel) as well as T47D cells with stable empty vector or ME1 expression (right panel). The level of glucose was shown in the bar graph (mean ± SD in three separate experiments). *p < 0.05 by Student’s t-test. (**d**) The content of lactate from cell lines in (**c**) were analyzed as described in the Materials and Methods. The level of lactate is shown in the bar graph (mean ± SD in three separate experiments). *p < 0.05 by Student’s t-test. (**e**) ECAR in SUM159 cells with stable empty vector or knockdown of ME1 expression (**a**) as well as T47D cells with stable empty vector or ME1 expression (**b**) was measured by Seahorse XF96 Extracellular Flux Analyzer (mean ± SD in three separate experiments).

**Figure 4:**
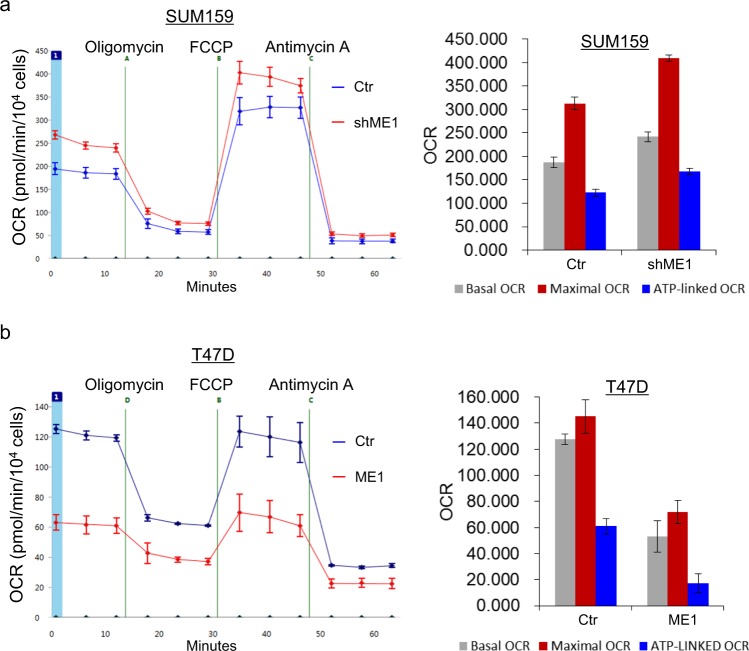
ME1 inhibits cell oxygen consumption. (**a** and **b**) Oxygen consumption in SUM159 cells with stable empty vector or knockdown of ME1 expression (**a**) as well as T47D cells with stable empty vector or ME1 expression (**b**) was measured by Seahorse XF96 Extracellular Flux Analyzer (mean ± SD in three separate experiments).

To explore the potential molecular function and mechanism of ME1, we generated stable transfectants with empty vector or knockdown of ME1 expression in SUM159 cell and clones with empty vector or ME1 expression in T47D cells (Fig. 3b and Supplementary Fig. 2). Most cancer cells, unlike their normal counterpart, predominantly consume more glucose by Warburg effect. As ME1 links glycolysis and citric acid cycle (Fig. 3a), we investigated whether ME1 expression contributes to the metabolic alteration. We first examined glucose uptake. We observed that ME1 expression significantly enhanced glucose uptake in T47D cells, whereas knockdown of ME1 expression decrease glucose uptake in SUM159 cells (Fig. 3c). We then measured lactate production and found that ME1-expressing T47D cells produced more lactate than their vector control cells, whereas ME1-knockdown SUM159 cells had less lactate production (Fig. 3d). We also measured extracellular acidification rates (ECAR), showing that ME1 expression had an increased ECAR in T47D cells, whereas knockdown of ME1 expression led to decreased ECAR in SUM159 cells (Fig. 3e). Oxygen is necessary for ATP synthesis by OXPHOS. We next detected oxygen consumption rate (OCR). Noticeably, knockdown of ME1 expression in SUM159 cells resulted in a great increase in basal OCR (Fig. 4a), whereas exogenous ME1 expression in T47D cells displayed a significant decrease in the basal OCR compared with the vector control (Fig. 4b). Similar results were obtained in the analysis of ATP-linked and maximal OCR (Fig. 4). These data indicate that ME1 attenuates OXPHOS and promotes aerobic glycolysis.

### ME1 promotes tumor cell growth under hypoxic condition and colony-formation

**Figure 5:**
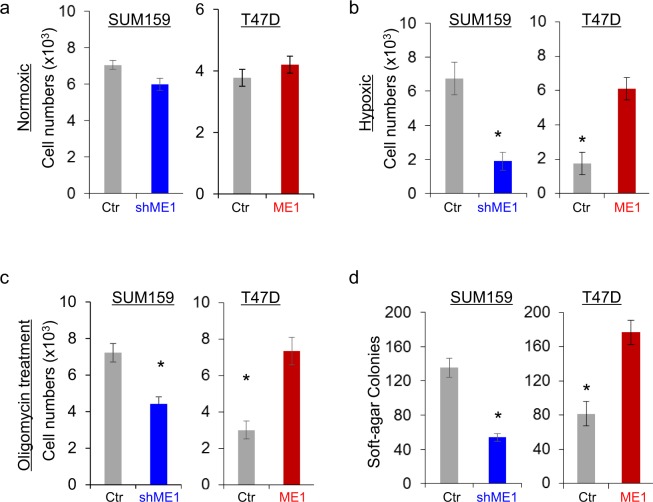
Knockdown of ME1 inhibits cell growth under hypoxia and reduces colony-formation. (**a**) Cell growth under normoxic condition for SUM159 cells with stable empty vector or knockdown of ME1 expression (left panel) as well as T47D cells with stable empty vector or ME1 expression (right panel) was measured by cell-count assay for a period of 3 days. Statistical analyses are plotted (mean ± SD in three separate experiments). (**b**) Cell growth under hypoxic condition was measured as in (**a**). Statistical analyses are plotted (mean ± SD in three separate experiments). *p < 0.01 by Student’s t-test. (**c**) Cell growth following treatment with or without oligomycin (25 nM) under hypoxic condition was measured as in (**a**). Statistical analyses are plotted (mean ± SD in three separate experiments). *p < 0.01 by Student’s t-test. (**d**) The formation of colonies from cells in (**a**) was measured. Statistical analyses are plotted (mean ± SD in three separate experiments). *p < 0.01 by Student’s t-test.

Given the observation that ME1 expression decrease oxygen consumption, we evaluated the effects of ME1 on cell growth under different oxygen conditions. Under normoxic condition (21% O2), knockdown of ME1 expression in SUM159 cells or exogenous ME1 expression in T47D cells only caused a minor change in cell growth (Fig. 5a). Interestingly, under hypoxic condition (0.1% O2), knockdown of ME1 expression caused a remarkable decrease in cell growth in SUM159 cells, whereas ME1 expression dramatically increased cell proliferation in T47D cells Fig. 5b. We also evaluated the effect of oligomycin, an inhibitor of ATP synthase that is used to prevent state 3 respiration on cell proliferation. Consistent with hypoxia, following treatment of oligomycin, knockdown of ME1 expression in SUM159 induced a remarkable inhibition in cell growth whereas ME1 expression in T47D cells significantly increased cell proliferation (Fig. 5c). These findings indicate that tumor cell with loss of ME1 expression are more oxygen-dependent, whereas ME1 expression leads to adaptation of tumor cells to hypoxic environment.

To characterize the effect of ME1 expression on the *in vitro* tumorigenicity, the cells were examined by soft-agar assay. Intriguingly, knockdown of ME1 expression decreased colony-formation in SUM159 cells, whereas exogenous ME1 expression exhibited higher capability to form colonies in T47D cells (Fig. 5d).

### ME1 contributes to tumorigenicity and predicts poor prognosis

**Figure 6:**
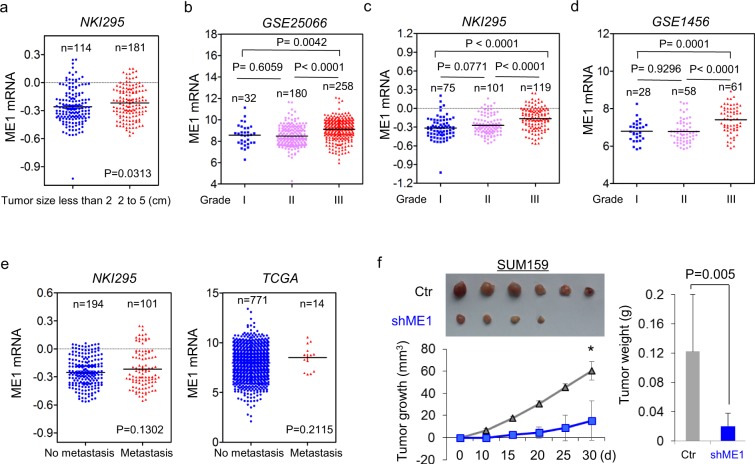
Knockdown of ME1 suppresses tumorigenicity. (**a**) Scatter dot-plot indicated ME1 expression in different tumor size of breast cancer from NKI295 dataset. (**b**–**d**) Scatter dot-plots indicated ME1 expression in three histological grades of breast cancer from GSE25066 (**b**), NKI295 (**c**) and GSE1456 (**d**) datasets. (**e**) Analysis of the relationship between ME1 expression and metastasis. (**f**) SUM159 cells with stable empty vector or knockdown of ME1 expression were injected into the mammary fat pad of female SCID mice. The growth of breast tumors was monitored. Mice were sacrificed after 30 days. Tumor size and weight were measured and recorded. Data were presented as mean ± SEM from six mice.

To identify a possible association between ME1 expression and clinical oncology, we first assessed the correlation between ME1 expression and tumor size of breast cancer patients in NKI295 dataset. Patients were separated into two groups according to tumor size of patients. As anticipated, high ME1 expression was associated with a large tumor size of breast cancer patients (Fig. 6a). We then analyzed the relationship between ME1 expression and histological grades of the tumors in GSE25066, NKI295, and GSE1456 datasets in which tumors had been scored for tumor grade. We segregated patients into three groups according to histological grades of tumors. Intriguingly, ME1 expression was present predominantly in Grade 3 tumors but far less commonly in Grade 1 and Grade 2 tumors Fig. 6b–d. These data confirmed the notion that ME1 is an important mediator of BLBC progression.

Although ME1 overexpression highly correlated with larger tumor size and higher tumor grade in breast cancer patients, it didn’t significantly affect metastatic status of breast cancer through analyzing the correlation between ME1 expression and metastatic status in NKI295 and TCGA datasets (Fig. 6e). We thus determined the effect of ME1 expression on the *in vivo* tumorigenicity. We performed tumor xenograft experiment in which the mammary fat pads of female SCID mice were injected with SUM159 cells with stable empty vector or knockdown of ME1 expression. As shown in Fig. 6f, SUM159 cells with stable knockdown of ME1 expression had dramatically reduced tumor growth compared with their corresponding vector cells, suggesting that ME1 is critical for tumorigenicity of BLBC cells.

**Figure 7:**
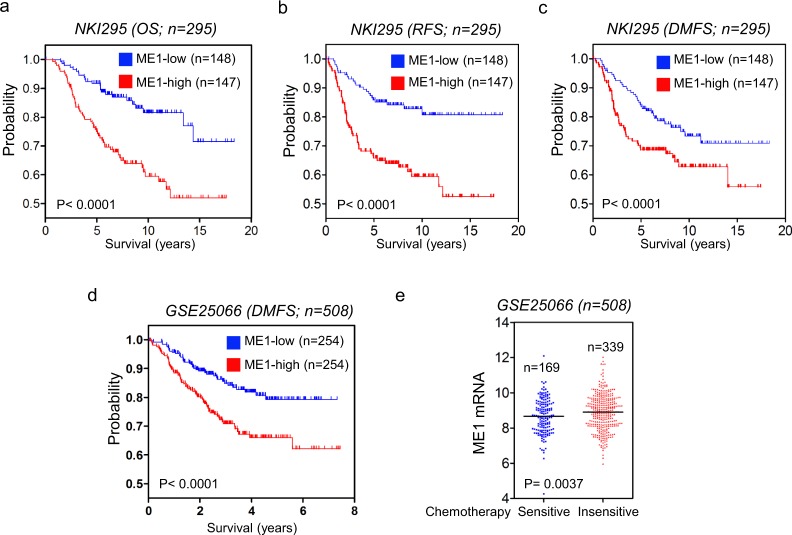
ME1 expression predicts poor clinical outcome. (**a**–**c**) Kaplan-Meier survival analysis for OS (**a**), RFS (**b**) and DMFS (**c**) of patients in the NKI295 dataset according to ME1 expression status. The p value was presented using the log-rank test. (**d**) Kaplan-Meier survival analysis for DMFS of patients in the GSE25066 dataset according to ME1 expression status. The p value was presented using the log-rank test. (**e**) Analysis of GSE25066 dataset for the relationship between ME1 expression and chemotherapy sensitivity.

Given the critical roles of ME1 expression in breast cancer, we performed Kaplan-Meier analyses to evaluate whether ME1 is a prognostic indicator for clinical outcomes by analyzing NKI295 and GSE25066 datasets^22,23^. We segregated patients into two groups according to ME1 expression, with high ME1 expression having poorer overall (OS), relapse-free (RFS), and distant metastasis-free survival (DMFS) (Fig. 7a–d). We then assessed whether ME1 expression was correlated with chemotherapy sensitivity in GSE25066 dataset in which patients were treated with sequential taxane and anthracycline–based regimens. Apparently, elevated ME1 expression is associated with chemotherapy resistance (Fig. 7e). These clinical validations support the potential use of ME1 as a newly prognostic indicator and therapeutic target for breast cancer patients.

## Discussion

ME1 provides metabolic and tumorigenic advantages in BLBC cells. Our study provides several insights into the vital roles of ME1 in BLBC.

### ME1 provides BLBC cells with metabolic plasticity

The aerobic glycolysis is a vital component of the metabolic reprogramming^10–12^. Our study showed that ME1 expression resulted in the metabolic alteration in BLBC cells. These include: 1) increase of glycolysis by increasing glucose uptake and glycolytic flux. The transition from premalignant lesions to cancer is often accompanied by increased tumor glucose uptake and lactate production^28^. Our study identified that knockdown of ME1 expression impaired glucose uptake and reduces lactate generation in BLBC cells. 2) suppression of OXPHOS. Decreased aerobic capacity is a universal feature of tumors^29,30^. Consistently, our results showed that ME1-silencing cells were more oxygen-dependent. Thus, the alteration of cellular metabolism induced by ME1 expression is in concordance with the characteristic of the aerobic glycolysis. In addition, we observed that BLBC cell line SUM159 cells with endogenous ME1 expression have stronger adaptation to hypoxia than luminal cell line T47D cells with low endogenous ME1 expression (Fig. 5a,b), suggesting ME1 may facilitate BLBC cells to be better suited to cope with metabolic challenges. Indeed, we demonstrated that ME1 expression promoted, whereas knockdown of ME1 attenuated adaptation of tumor cells to hypoxic environment. Together, these data suggest that ME1 provides BLBC cells with metabolic plasticity.

### ME1 upregulation associates with copy number amplification of ME1 gene and promotes tumorigenicity of BLBC cells

CNVs play a critical role in tumor susceptibility. Upregulation of ME1 expression may associate with an increase in copy number at the genomic locus containing ME1. In this study, we identified ME1 as a frequently amplified gene by analyzing CNVs in two large datasets from breast cancer tissues (MEBTABRIC and TCGA) and patient-derived breast cancer cell lines (CCLE), and found that cases with ME1 copy number amplification had much higher ME1 expression than ones with no amplification, supporting that ME1 copy number amplification positively correlates with ME1 overexpression in breast cancer. Consistently, in most cases copy number amplification in ME1 and its resultant ME1 overexpression were positively associated with BLBC.

It has been identified that the aerobic glycolysis promotes the tumorigenicity of cancer cells^31,32^. Our soft-agar assay showed ME1 expression contributed to colony-formation of breast cancer cells, indicating that ME1 might promote a more stem-like phenotype. Tumor cells with stem cell-like properties are essential for drug resistance^33–35^. Indeed, high ME1 expression is positively associated with chemotherapy resistance (Fig. 7e). In the clinic, aberrant high ME1 expression is specifically observed in BLBC subtype, and highly correlates with larger tumor size and higher grade in breast cancer patients. In line with this notion, knockdown of ME1 expression suppresses tumorigenicity of BLBC in the mouse model, indicating that ME1 serves as a key metabolic hub in BLBC progression.

### Our study provides a potential prognostic marker and molecular target for BLBC

Our study identifies that ME1 expression links cancer progression and patient prognosis, including: (1) ME1 expression is dramatically upregulated in breast cancer with basal-like subtype; (2) Elevated ME1 expression is highly correlated with larger tumor size; (3) Elevated ME1 expression is significantly associated with higher grade; (4) Elevated ME1 expression is associated with shorter overall, relapse-free, and distant metastasis-free survival; (5) ME1 expression is negatively correlated with treatment outcome in breast cancer patients. These findings clearly suggest that ME1 may become a newly useful prognostic indicator for breast cancer patients. Treatment of BLBC is an unmet medical need due to the absence of effective targeted agents. Thus, identification of the novel targets in BLBC will promote the development of new treatment strategy against this disease. Our study identified the critical roles of ME1 in metabolic reprogramming and BLBC. Thus, blocking ME1 expression might contribute to the application of therapeutic intervention for BLBC. Together, our study indicates that ME1 is a novel prognostic indicator for BLBC, and may become a valuable molecular target for treating BLBC.

## Materials and Methods

### Plasmids, shRNA, and Antibodies

ME1 shRNA was purchased from MISSION shRNA at Sigma-Aldrich (St Louis, MO). Human ME1 was amplified from a SUM159 cDNA library and subcloned into pBABE. Antibodies against ME1 and β-actin were purchased from Sigma-Aldrich (St. Louis, MO).

### Cell Culture

SUM159 cells were grown in DMEM plus 10% fetal bovine serum (FBS). T47D cells were grown in RPMI1640 supplemented with 10% FBS. For establishing stable transfectants with ME1 expression or knockdown of ME1 expression, T47D cells and SUM159 cells were transfected with pBABE-ME1 and ME1 shRNA, respectively; stable clones were selected with puromycin (200 ng/ml) for 3–4 wks.

### Western blotting of tumor samples

The tumor samples were homogenized by 20 strokes of a Dounce homogenizer in 1 ml of homogenizing buffer. Following centrifugation, the pellets were collected, re-suspended and boiled in Laemmli buffer. The proteins were analyzed by SDS-PAGE, and then transferred onto PVDF membranes (Thermo Fisher Scientific). Immunoreactive blots were visualized by chemiluminescence.

### Metabolism Assay

Glucose uptake and lactate production were measured by glucose assay kit (BioVision) and lactate assay kit (BioVision), respectively. Real-time basal oxygen consumption (OCR) was determined using the Seahorse Extracellular Flux (XF-96) analyzer (Seahorse Bioscience, Chicopee, MA). The XF-96 measures the concentration of oxygen in the medium above a monolayer of cells in real-time. Thus, the rates of oxygen consumption can be measured across several samples at a time. To compare between experiments, data are presented as the rate of oxygen consumption rate (OCR) or extracellular acidification rates (ECAR) in pMoles/min/10^4^ cells or mpH/min/10^4^ cells. Basal OCR or ECAR was examined four times and plotted as a function of cells with and without treatment under the basal condition followed by the sequential treatment of oligomycin (1 μg/ml), FCCP (1 μM) and Antimycin A (5 μM). This progress curve displays the relative contribution of non-respiratory-chain oxygen consumption, ATP-linked oxygen consumption, the maximal OCR after the FCCP addition, and the reserve capacity of the cells.

### Colony formation assay

Colony formation was assayed using double-layer soft agar in 6-well plates with a bottom layer of 0.7% agar and a top layer of 0.35% agar. Cells were seeded into 24-well plates in desired medium and cultured at 37 °C for 2 to 3 weeks, and the colonies were stained and counted.

### Xenograft studies

The mice were injected with 1 × 10^6^ cells ME1-knockdown cells on the right flank and vector control cells on the left flank. Tumor growth was measured every 2 to 3 days for 30 days. The tumors were harvested at the experimental endpoint, and the masses of tumors derived from cells with ME1-knockdown and vector control in both flanks of each mouse were compared. Data were analyzed using the Student’s t-test; a p value < 0.05 was considered significant.

### Statistical Analysis

Results are shown as mean + SD or SEM as indicated. The two-tailed Student’s t-test, or one-way ANOVA was used to compare the intergroup. Survival curves were plotted using the Kaplan-Meier method, and differences were analyzed with the log-rank test. In all statistical tests, p < 0.05 was considered statistically significant.

### Ethical Statement

The tumor samples were collected from resected breast tumors from patients with informed consent. The experiments were performed according to the approved guidelines established by the institutional review board at the Zhejiang University, Hangzhou, China. Animal experiments were performed according to procedures approved by the Institutional Animal Care and Use Committee at the Zhejiang University, Hangzhou, China.

## Electronic supplementary material

Supplementary Figures
