# Supplementary material for: ME1 promotes basal-like breast cancer progression and associates with poor prognosis
Source: Sci Rep. 2018 Nov 13;8:16743. doi: 10.1038/s41598-018-35106-y (PMC6233160; doi:10.1038/s41598-018-35106-y)
Supplement: Supplementary file 1 — Supplementary Figures [file 41598_2018_35106_MOESM1_ESM.doc]

**ME1 promotes basal-like breast cancer progression and associates with poor prognosis**

Ruocen Liao1, 2**‡**, Guoping Ren1, 3**‡**, Huixin Liu1, 2**‡**, Xingyu Chen1, 2, Qianhua Cao1, 2, Xuebiao Wu1, 2，Jun Li4 and Chenfang Dong1, 2­

**Supplementary Figures**


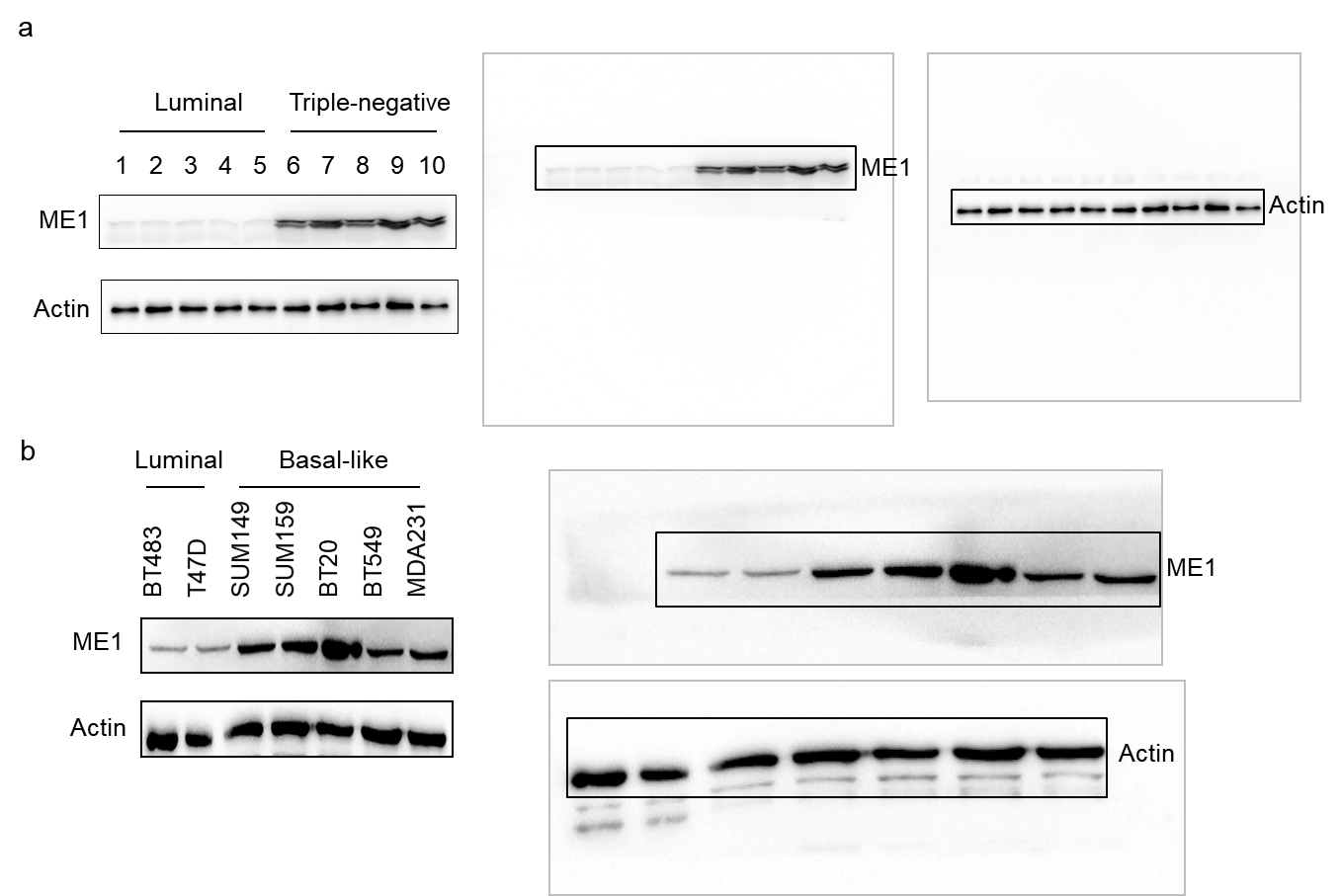


**Supplementary Figure 1** ME1 expression of tumor samples and cell lines as analyzed by western blotting. (a) Expression of ME1 was examined by western blotting in tumor samples from five cases of luminal and five cases of triple-negative breast cancer. (b) Expression of ME1 in two luminal and five BLBC cell lines was analyzed by western blotting. Uncut gels were shown in the right panel.


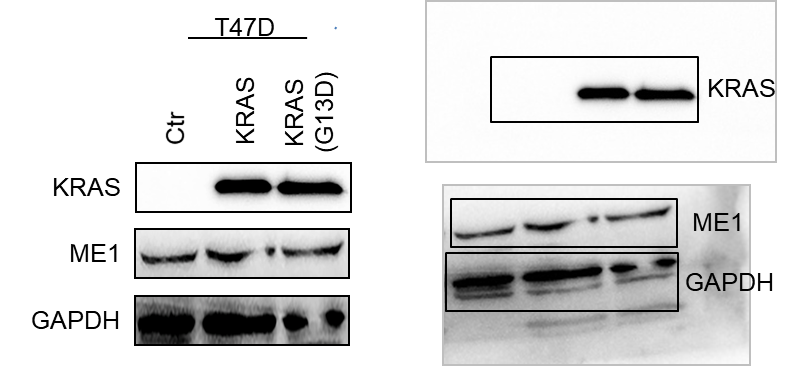


**Supplementary Figure 2** ME1 expression was analyzed by western blotting in T47D cells transfected with empty vector, KRAS-expressing vector or oncogenic KRAS (G13D)-expressing vector. Uncut gels were shown in the right panel.


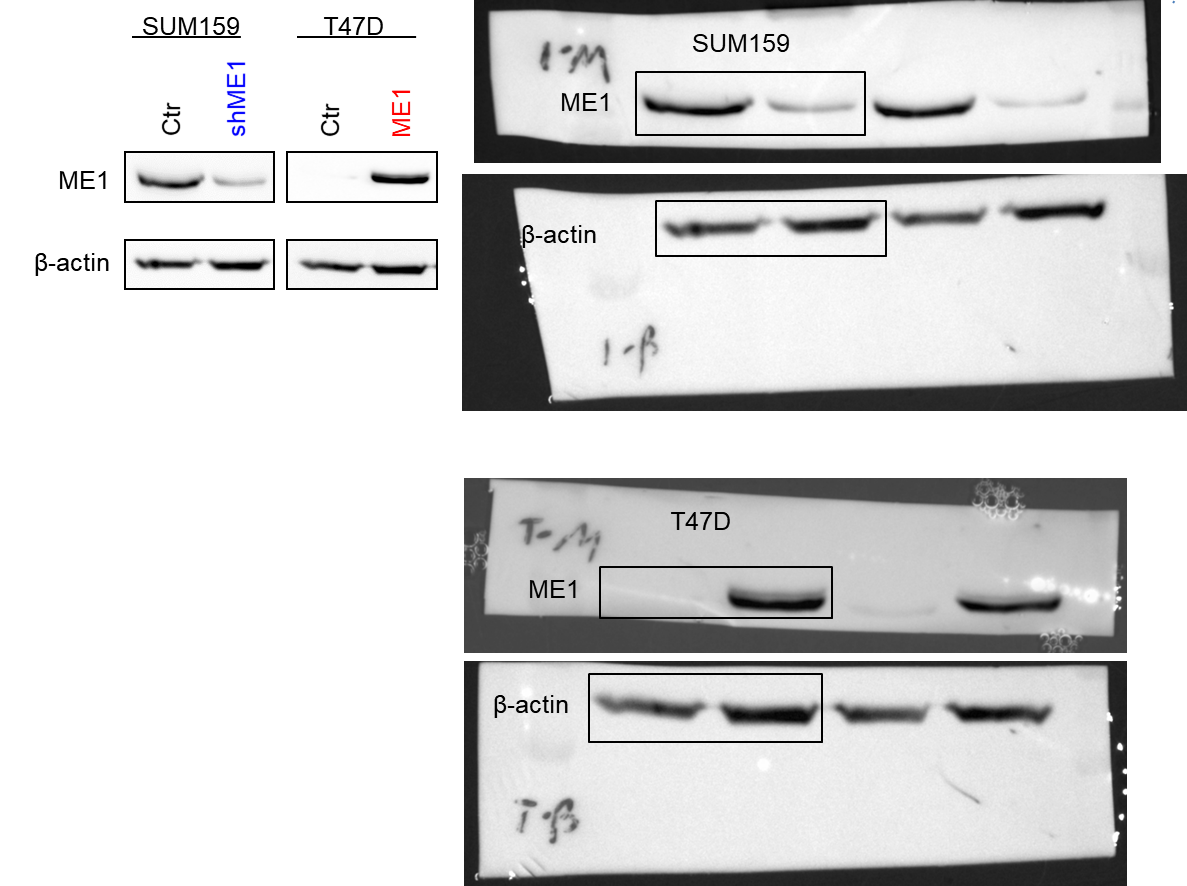


**Supplementary Figure 3** ME1 expression of cells as analyzed by western blotting. Stable transfectants with empty vector or knockdown of ME1 expression were established in SUM159 cells, and stable clones with empty vector or ME1 expression were generated in T47D cells. ME1 expression in these cells was examined by western blotting. Actin was used as a loading control. Uncut gels were shown in the right panel.
